# Brucellosis unusually presented as septic knee arthritis: A case report

**DOI:** 10.1002/ccr3.6461

**Published:** 2022-10-13

**Authors:** Bassem Al Hariri, Mohanned Zuhair, Abdulqadir J. Nashwan

**Affiliations:** ^1^ Internal Medicine Department Hazm Mebaireek General Hospital, Hamad Medical Corporation Doha Qatar; ^2^ Nursing Department Hazm Mebaireek General Hospital, Hamad Medical Corporation Doha Qatar

**Keywords:** brucella melitensis, brucellosis, knee septic arthritis, osteoarticular involvement

## Abstract

Brucellosis is one of the world's most prevalent zoonotic illnesses. The most often afflicted joints are the sacroiliac joints, although spondylitis and peripheral arthritis are becoming increasingly prevalent. We described a case of a 40‐year‐old male patient with Brucellosis presented as septic knee arthritis.

## Graphical abstract

All patients with septic arthritis who reside in endemic areas should be screened for Brucellosis as appropriate.

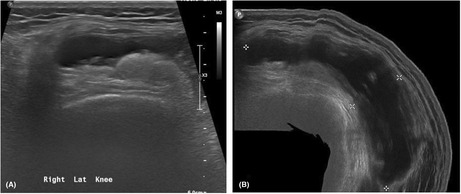

## 1 INTRODUCTION

Brucellosis is one of the world's most prevalent zoonotic illnesses,^1^, ^2^ especially in the East Mediterranean region (EMRO).^3^ The most common brucellosis sign is osteoarticular involvement. The most often afflicted joints are the sacroiliac joints, although spondylitis and peripheral arthritis are becoming increasingly prevalent. Brucellosis is highly vulnerable to being ignored in the presence of companion bacteria.^3^ As a consequence, it should be considered in all individuals with septic arthritis who reside or travel through endemic areas.

## 2 CASE PRESENTATION

**FIGURE 1:**
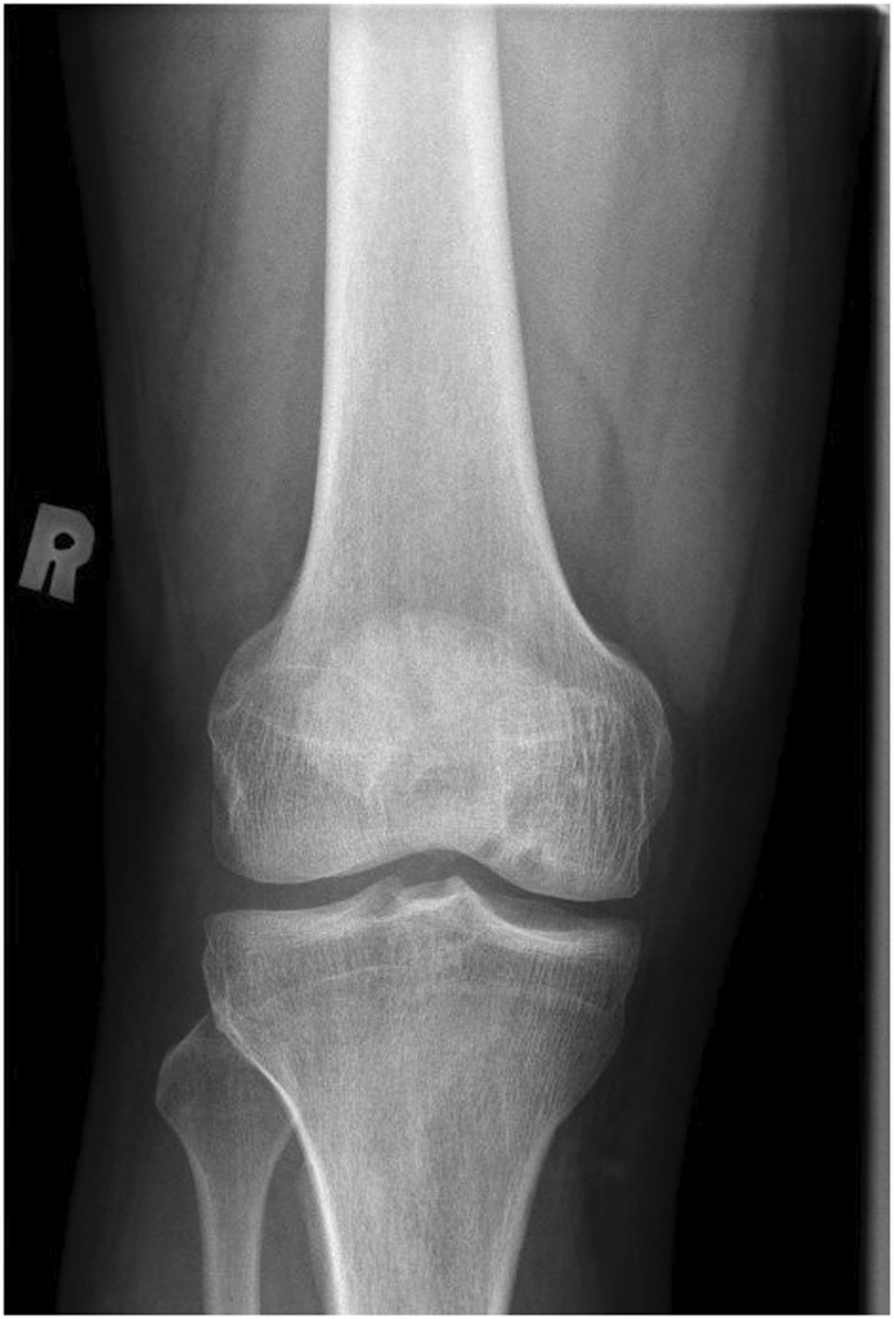
XR Right Knee (unremarkable)

**FIGURE 2:**
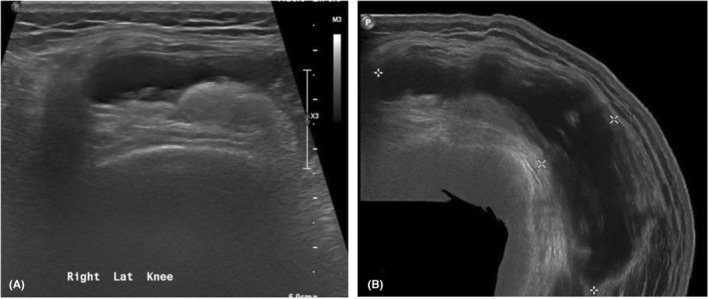
US Soft Tissue Right Knee. (A) Large joint effusion is noted reaching the suprapatellar bursa associated with synovial irregular thickening and hypervascularity for clinical correlation to rule out septic arthritis. (B) The largest collection is measuring 8.1 × 2.2 cm

A 40‐year‐old male patient with no prior medical history was hospitalized for 7 days due right knee pain and swelling. Fever has been around for the past 2 weeks, coming and going. The discomfort made it impossible for him to walk. There were no gastrointestinal or genitourinary symptoms and unremarkable cardiopulmonary and neurological review. No other joint involvement. No skin rashes and there was no history of trauma. The patient stated that he had a similar episode of knee pain 10 years ago. He denied any contact with animals and no history of raw milk consumption. His temperature was 37.8°C. The (R) knee was swollen, hot, and tender with a limited range of movement (Figure 1). The deferential diagnosis included septic arthritis and crystal‐induced arthritis. The general examination was normal. Laboratory investigation showed white blood count (WBC) of 5.2 and C‐Reactive Protein (CRP) was high 110. Hemoglobin (Hb) was 12.9, mean corpuscular volume (MCV) 60.9; red blood cell distribution width (RDW) was 18.6, suggestive of iron deficiency anemia. Platelets were normal at 270. The coagulation profile showed high prothrombin time (PT) 12.8, international normalized ratio (INR), and partial thromboplastin time (PTT) were normal. Blood chemistry investigations were all normal. Brucella serology was done, which showed Brucella IgG was positive 1: 320, and IgM was negative. Synovial fluid aspirate revealed yellow, turbid fluid with WBCs of 4800, red blood cells (RBCs) 13,475, neutrophils 31.0, lymphocytes 2.0, and monocytes 67. *Brucella melitensis* was detected in both blood cultures and synovial fluid (This organism has presumptively been identified as Brucella species). Brucella fall into WHO risk group. The echocardiogram did not reveal vegetation. Right knee soft tissue ultrasound revealed large joint effusion reaching suprapatellar bursa associated with synovial irregular thickening and hypervascularity (Figure 2). The largest collection measuring 8.1 × 2.2 cm. He was prescribed gentamycin 5 mg/kg once IV for 7 days. In addition, doxycycline 100 mg and rifampin 450 mg twice a day for 12 weeks. A follow‐up appointment was arranged 2 weeks after discharge, patient reported mild limping without pain, but the knee joint was still swollen as compared to the other joints. He was given another appointment 1 month later which showed that his condition has improved.

## 3 DISCUSSION

One of the most widespread zoonotic diseases is brucellosis.^4^ Dairy (unpasteurized) products, diseased animals' close contact, or the products of an animal's conception can all lead to infection. Rare instances of mother‐to‐child transmission of brucellosis during pregnancy and breastfeeding have been documented.^5^ Because of its high infectivity by inhalation, brucellosis has the potential to be used as a biological weapon.^6^

Infections with *B. melitensis* continue to be an urgent public health concern in Mediterranean nations, southern, central, and western Asia, as well as certain portions of South and Central America, and many African countries. More than 100/100,000 person‐years have been documented in the Middle East region, including Jordan, Iraq, and KSA.^7^, ^8^ Despite appearing to be on the decline in some regions of Saudi Arabia, brucellosis is nevertheless an endemic disease, with prevalence peaking in those between the ages of 40 and 49.^9^

Brucellosis patients frequently experience a variety of symptoms, including splenomegaly, arthralgia, high fever, and malodorous perspiration. In some situations, the onset may be gradual or there may be a predominance of one organ over others (focal brucellosis). One of the brucellosis symptoms that occurs most frequently is osteoarticular involvement. There have been reports of peripheral arthritis, spondylitis, sacroiliitis, bursitis, and osteomyelitis among other conditions. The wrist and ankle tendons are the most frequently affected, and tenosynovitis comparable to the first documented case in 1908.^10^ In a relatively recent meta‐analysis, 26% of the infected individuals had arthritis, compared with 65% of patients with arthralgia. Spondylitis and sacroiliitis were seen in 12% to 36% of individuals overall.^11^

Especially in nonendemic locations, brucella arthritis diagnosis might be difficult. In endemic locations, serology—often in conjunction with conventional agglutination tests (SAT) is the mainstay of diagnosis.^8^ Blood cultures often require a long incubation period and have variable sensitivity ranging from 53% to 90%. Blood cultures may come back negative when the disease is limited to a single joint, so serology continues to serve as the foundation for laboratory diagnosis. Despite negative blood cultures, synovial fluid cultures can nonetheless be positive.

Blood culture sensitivity has increased with the use of automated systems, reaching up to 95%, while incubation times have decreased to just 7 days, (93.3%, 14/15) BACTEC cultures, (75.0%, 6/8) isolator cultures, and (57.1%, 4/7) conventional cultures all supported *B. melitensis* growth.^12^ Leukocyte counts in synovial fluid analysis often show an exudative process with values between a few hundred and a few thousand.^2^

Although the two can occasionally coexist, synovial fluid analysis aids in differentiating crystal arthropathy from viral arthritis.^13^ Although these approaches have decreased incubation, a faster and more accurate analysis is still required. Compared with conventional methods, PCR has demonstrated great sensitivity and specificity, enabling quicker and more accurate identification of the Brucella. However, because of issues with standards, its use is still infrequent.^14^ Recent study has shown that MALDI‐TOF MS is a simple, rapid, and highly accurate approach for identifying brucella.^15^

Because monotherapy is associated with significant recurrence rates, a two‐drug combination is used. When compared with combination treatment, monotherapy had more than double the probability of overall failure (relative risk, 2.56).^16^ The recommended course of treatment includes doxycycline 100 mg twice daily for six weeks and injectable streptomycin 0.75–1 gm once a day for a maximum of 3 weeks. In cases of serious brucellosis, triple therapy for a course lasting longer than 3 months is advised. With a shorter period of fewer than 6 weeks, both treatment failure (3.02, 1.03–8.80) and relapse (1.70, 1.19–2.44) were substantially more frequent. Contrarily, the aminoglycoside/doxycycline combination had a lower relapse rate than the combination of rifampicin and doxycycline, especially in cases of osteoarticular disease.^16^

According to a meta‐analysis, 5%–7% of patients treated with doxycycline‐streptomycin and 11%–17% with doxycycline‐rifampin experienced treatment failure or relapse.^8^ The simultaneous administration of rifampicin and doxycycline may have lowered the blood level of the drug, which could be one explanation.^7^ Rifampicin resistance was not proven by molecular detection techniques or in vitro susceptibility tests. Overall, this low success rate is more likely attributable to poor compliance or a lack of time than to rifampicin resistance. Except for co‐trimoxazole, other drugs' minimum inhibitory concentrations remain reassuringly low. Notably, avoiding rifampicin will eliminate the possibility of inducing resistance in tuberculosis in regions where TB and brucellosis are both widespread, particularly when TB is misdiagnosed as the underlying cause.

Brucella infection in arthroplasties is a rare event. Unspecific clinical symptomatology is associated with unclear radiographic peri‐prosthetic signs of bone halisteresis. Only a positive anamnesis, combined with an antibiogram of the joint liquid and a high serum antibody titer, can lead to a definitive diagnosis. We report a case of *Brucella melitensis* infection in a total knee arthroplasty implanted 2 years earlier. With the absence of radiological signs of prosthetic loosening and thanks to a systemic antibiotic combined therapy (rifampicin + doxycycline) extended for 8 weeks, we were able to solve the infection by avoiding surgery.^17^

Another case was reported for a 63‐year‐old female patient who underwent a total knee arthroplasty in which the knee later became infected with *Brucella melitensis*. The diagnosis was made by positive culture of a sinus tract discharge. Radiological views of the knee did not show signs of implant loosening. The patient was successfully treated with rifampicin and doxycycline without surgery.^18^

Compared with septic arthritis brought on by pyogenic organisms, *B melitensis* patients experience modest joint inflammation, and erythema of the overlying skin is rare.^10^ Having said that, making a clinical diagnosis based solely on local inflammatory symptoms may be challenging. The most common sign of brucellosis that should notify a doctor is a fever, which is typically undulant.

## 4 CONCLUSION

Brucellosis is still a difficult disease in endemic regions. Its many rheumatologic symptoms may be mistaken for various forms of arthritis. In non‐endemic locations, extensive travel and contact histories are required to make an early diagnosis. In such circumstances, blood, synovial cultures, and brucellosis serology should be performed. Antibiotics combos and a lengthy duration of therapy are required to avoid brucella septic arthritis failure or recurrence.

## AUTHOR CONTRIBUTIONS

BAH, MZ, and AJN performed data collection, literature search, and manuscript preparation. All authors read and approved the final manuscript.

## CONFLICT OF INTEREST

The authors declare that they have no competing interests.

## ETHICAL APPROVAL

The article describes a case report. Therefore, no additional permission from our Ethics Committee was required.

## CONSENT

Written informed consent was obtained from the patient to publish this report in accordance with the journal's patient consent policy.

## Data Availability

All data generated or analyzed during this study are included in this published article.
